# Bidirectional Interactions With Humpback Whale Singer Using Concrete Sound Elements

**DOI:** 10.3389/fpsyg.2021.654314

**Published:** 2021-06-11

**Authors:** Aline Pénitot, Diemo Schwarz, Paul Nguyen Hong Duc, Dorian Cazau, Olivier Adam

**Affiliations:** ^1^Compagnie Ondas, Paris, France; ^2^Sciences et Technologies de la Musique et du Son, UMR 9912, Ircam, CNRS, Ministère de la Culture, Sorbonne Université, Paris, France; ^3^CNRS, Institut Jean Le Rond d’Alembert, UMR 7190, Sorbonne Université, Paris, France; ^4^ENSTA Bretagne, CNRS, Lab-STICC, UMR 6285, Brest, France; ^5^CNRS, Institut des Neurosciences Paris-Saclay, UMR 9197, Université Paris-Saclay, Orsay, France

**Keywords:** humpback whale song, bassoon, interactions, music interface, musique concrete

## Abstract

We describe an art–science project called “Feral Interactions—The Answer of the Humpback Whale” inspired by humpback whale songs and interactions between individuals based on mutual influences, learning process, or ranking in the dominance hierarchy. The aim was to build new sounds that can be used to initiate acoustic interactions with these whales, not in a one-way direction, as playbacks do, but in real interspecies exchanges. Thus, we investigated how the humpback whales generate sounds in order to better understand their abilities and limits. By carefully listening to their emitted vocalizations, we also describe their acoustic features and temporal structure, in a scientific way and also with a musical approach as it is done with *musique concrète*, in order to specify the types and the morphologies of whale sounds. The idea is to highlight the most precise information to generate our own sounds that will be suggested to the whales. Based on the approach developed in *musique concrète*, similarities with the sounds produced by bassoon were identified and then were processed to become “concrete sound elements.” This analysis also brought us to design a new music interface that allows us to create adapted musical phrases in real-time. With this approach, interactions will be possible in both directions, from and to whales.

## Introduction

Intra- or interspecies interactions are the effects between individuals respectively from the same or different species, based on sensory stimuli from at least one of their senses including sight, smell, touch, taste, and hearing (Cheng, 1991). Durations of these interactions have different levels of consequences on their life and vital activities (Wootton and Emmerson, 2005) and could be largely different from simple to complex interactions with positive associations (predator–prey relationships, symbiotic relationships such as mutualism, commensalism, and parasitism) and negative associations (competition). Cetaceans are highly social species living in groups. Intraspecies interactions are strong during their activities and strengthen the structure of their societies (see, for example, MacLeod, 1998; Orbach, 2019). However, numerous observations were also already done between different cetacean species. Observations of these interspecies interactions published in scientific revues mostly involved delphinids (Weller et al., 1996; Frantzis and Herzing, 2002; Herzing et al., 2003; Psarakos et al., 2003; Smultea et al., 2014). Interactions between odontoceti and mysticeti species seems rarer, sometimes in a relationship as prey/predator (Whitehead and Glass, 1985; Arnbom et al., 1987; Vidal and Pechter, 1989; Jefferson et al., 1991; Palacios and Mate, 1996; Rossi-Santos et al., 2009; Deakos et al., 2010; Cotter et al., 2012) or as competitors for food (Wedekin et al., 2004).

Humans were always interested in nature and their inhabitants, even when marine wildlife was more difficult to observe and to be in contact with, in spiritual and practical ways. In occidental civilizations, over centuries, cetaceans were shown like scary creatures with deformed imaginary bodies. In other parts of the world, cetaceans were considered gods and were revered (Constantine, 2002). More recently, during the 19th and 20th centuries, whales were massively hunted for food and goods. After the international whale moratorium in 1982, interactions with cetaceans were still negative, due to the numerous marine anthropogenic activities, like fisheries, marine traffic, plastic, chemical and sound pollution, and climate change. Nevertheless, the public progressively started to interact differently with cetaceans, especially because of another intellectual view of understanding the oceans (Alpers, 1963; Cousteau and Diolé, 1972; Constantine, 2002; Neimi, 2010; Allen, 2014). It is interesting to note that the cetacean morphology, and not their emitted sounds, was one of the main criteria for positive or negative perceptions that humans have from cetaceans. However, since the easier democratic access for all to pleasure sail and motor crafts, and the new interests for marine life, and more largely for the ocean, meetings with cetaceans became not rare anymore, and then interactions were possible and happened more often (Parsons and Brown, 2017). Thus, positive and negative interactions were described when cetacean species respectively come to or go away from humans (Allen, 2014; Butterworth and Simmonds, 2017). Films, documentaries, and seaworlds have also implied, in their own way, a radical change in public attitudes and in particular a renewed interest for these marine mammals (Lavigne et al., 1999; Samuels and Tyack, 2000). Moreover, countries started a new management of the oceans; and international, regional, and national policies to protect cetaceans and their marine environment have been written and put in place, also contributing to higher respect for these species (Baur et al., 1999).

Research projects were done on cetacean species, to better understand their behaviors, their interactions, their habitats, and their migrations routes and also to better describe the potential effects of anthropogenic activities on them and their marine environment. These programs involved scientists with different skills, such as biology, ecology, ethology, genetics, acoustics, signal processing, mathematics, and computer science. In 2007, we started an international project in collaboration with the Malagasy Ngo Cetamada and the Department of Animal Biology of the University of Antananarivo, Madagascar. The main objective was to characterize the Southwestern Indian Ocean humpback whale population (individual identification, spatial distribution, and potential breeding hotspots). We also focused on the songs emitted by the male individuals with the idea to automatically classify their vocalizations. Because these songs are mainly emitted by males during the breeding seasons, they could have specific roles in mating and for reproduction, to attract females and/or to keep males away from each other (Winn and Winn, 1978; Tyack, 1981; Tyack and Whitehead, 1983; Helweg et al., 1992; Darling and Bérubé, 2001; Darling et al., 2006; see review in Cholewiak et al., 2013). From the detection and the extraction of the vocalizations, their songs were analyzed in order to characterize the internal structures of the successive phrases. Payne and McVay (1971) introduced the concept of units (also called song units), as harmonic or pulsed vocalizations emitted between two silences. Manual and automatic classifications of units are usually done from acoustic time and frequency features, such as duration, relative acoustic intensity, fundamental frequency, presence of harmonics, and shape of the Fourier spectrogram (Dunlop et al., 2007; Cholewiak et al., 2013). These units are organized in time sequences and repeated to form the song themes (Payne and McVay, 1971). During the last five decades, scientists studied these songs, and it was firstly shown that their evolutions are very slow from 1 year to another with the transformations of existing units, and the removal and the insertion of few new units in a cultural evolution (Payne et al., 1983; Payne and Payne, 1985; Allen et al., 2019; see review in Garland and McGregor, 2020). None of these changes reveal the functions of songs. Secondly, whales from the same ocean interact with each other by sharing the same themes, considered as regional dialects. In a few cases, songs changed faster, with new units introduced by humpback whales from another area, although in fact the mechanism is still being disentangled and may include song learning on migratory routes leading to their characterization as cultural revolution (Noad et al., 2000; Allen et al., 2019; Owen et al., 2019). However, different populations often emit different themes, as do whales within the same population across spans of several years.

In 1970, Payne published a vinyl album record with humpback whale song recordings (Payne, 1970). This album was played at the United Nations and at the Japanese House of Representatives to motivate governments and official authorities to stop whale hunting. Whale songs were also a source of inspiration for artistic works (for example, Collins, 1970; Hovhaness, 1970; Crumb, 1971; Bush, 1978; Cage, 1980; Nollman, 1982; Walker, 2001; Potter, 2003—see a review in Rothenberg, 2008a).

Another motivation was the goal to interact with a nonhuman species. Some of them are directly linked with vital activities, like territory defense or food search. Other interactions are based on mutual visual observations. With cetaceans, first experiments were focused on their abilities, their behaviors, and even their intelligence to manage such exchanges with humans. For example, a catalog of words was taught to dolphins (Lilly, 1967), showing that they can learn more than 30 words and can organize them in sentences (Herman et al., 1969; see review in Herman, 2010). Recently, this anthropomorphism approach was replaced by studies using what the cetaceans can do by themselves, as emitted sounds and behaviors (Dudzinski, 2010; Herzing, 2011; Reiss, 2011). For humpback whales, studies based on playback of their own emitted sounds helped to distinguish specific sounds like breeding or feeding sounds (Tyack, 1983; Mobley et al., 1988; Frankel and Clark, 1998; Darling et al., 2012; Dunlop et al., 2013).

Human music was also introduced during interactions; for example, composed and improvised musical pieces produced with flute, cello, violin, and even orchestra were played for dolphins, belugas, and humpback whales (for example, Video on YouTube, 2008, 2011a, 2011b, 2015). A small number of musical performances were also done by listening to sounds emitted by cetaceans during bidirectional interactions and already showed that these interactions were possible even with wild cetaceans. For example, Rothenberg (2008b) played clarinet to humpback whales in Hawaii, United States, and was able to adjust his musical improvisation taking account of the units emitted by the whale. Nollman (2008) played music with orcas in their marine environment. However, beside the musicians’ perceptions, no scientific method has been used to objectively characterize these interactions with whales.

Our art–science project “Feral Interactions—The Answer of the Humpback Whale” began in this context, with the motivation to take into account the humpback whale units and also to objectively measure the interaction level. Our first step was to carefully listen to the humpback whale songs following the analytic listening invented by Schaeffer (1967). Analytic listening is the foundation of *musique concrète*, which is not based on music sheets or on relationships between music notes and chords, or on personal perception by instrumentalists. It consists of selecting musical objects and analyzing the contents as well as their positions within a musical theme. Then, audio processing can be applied on these raw sounds to transform them into sound objects, finally totally disconnected from the musical instruments that produced the sounds. The aim is to assemble these objects to produce an original musical composition, which is played on loudspeakers. Schaeffer’s fundamental discovery was that these sound objects taken out of their context and used as musical objects are freed from the connotation of their source and can thus reveal their inherent sonic qualities.

Then, we identified some common acoustic features with sounds that can be obtained from a bassoon. We investigated the mechanical structure and the acoustic features of these two sound generators. In this paper, we will explain how our recent scientific study on the humpback whale larynx anatomy brought new inputs showing why the bassoon is the musical instrument closer to the vocal system of the humpback whales, rather than tapped or strummed string instruments or drums, even to simulate pulsating sounds. We will also present and analyze the results of a first experiment of human–animal interactions. Finally, we will discuss the design of a gestural interface for more nuanced and adaptive human–whale interaction.

## Similarities Between the Bassoon and the Humpback Whale Vocal Generator

Schaeffer (1966) highlighted the language of things and considered listening as the important first step, far from our cultural referents, to be able to create sound objects (Dufour et al., 1999). This process started from the origin of the sounds, i.e., what produced them but also on the perception and sensation of the listeners. It goes through “*écouter*” (indicative listening), “*ouïr*” (to be able to listen), before “*entendre*” (selective listening), and finally “*comprendre*” (identity listening) (Schaeffer, 1966). In this current study, we strictly followed these successive steps, beginning with the awareness of musical similarities between vocalizations emitted by humpback whales and sounds produced from bassoons. This “*trouvaille*,” as defined by Schaeffer (1966), is based on several common mechanical and acoustic features between the body of this musical instrument and the humpback whale vocal generator. Therefore, we were able to make comparisons between vibrators and resonators based on the features of the bassoon and our recent knowledge about the anatomy of the humpback whale larynx.

### Connections Between the Vibrators

**FIGURE 1:**
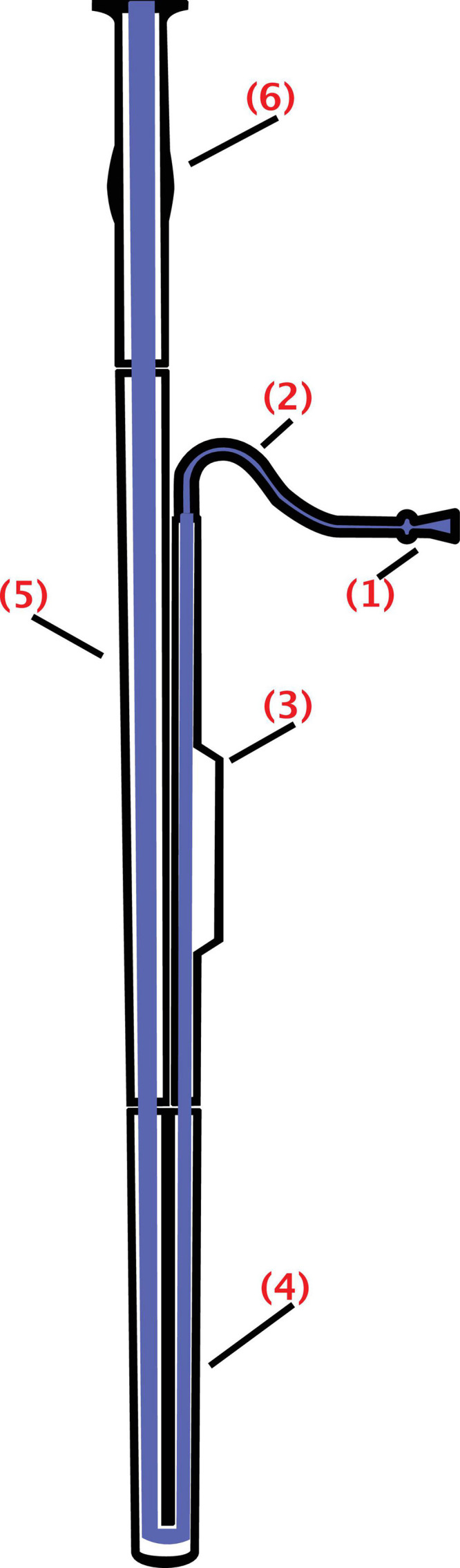
Schematics of the bassoon: The bell (6), extending upward; the bass joint (or long joint) **(5)**, connecting the bell and the boot; the boot (or butt) **(4)**, at the bottom of the instrument and folding over on itself; the wing joint (or tenor joint) **(3)**, which extends from boot to bocal; and the bocal (or crook) **(2)**, a crooked metal tube that attaches the wing joint to a reed **(1)** (from https://commons.wikimedia.org/wiki/File: Fagott-Bassoon.svg by users Mezzofortist, GMaxwell CC-BY-SA 3.0).

For the bassoon, the vibrator consists of two reeds tied together (Figure 1). The mouthpiece is thus made up of two parallel strips of delicately prepared and ligated reeds, unlike the clarinet and the saxophone, which have only one reed. Reeds are generally made from a plant call Arundo Donax. The long fibers are positioned from the heel up to the tip. A high quality of the reeds is absolutely needed to create sustainable sounds and not to produce unwanted whistles, generated by irregular facing (Brand, 1950; Giudici, 2019). These two reeds are flexible and mobile. Moistened and pinched between the lips of the instrumentalist, they vibrate with the airflow coming from the mouth. Periodic oscillations of the mouthpiece excite the air column (Nederveen, 1969). The fundamental frequency and the harmonics will depend on different parameters including the stiffness of the two reeds, the pressure of the lips, the power of the airflow, the length of the air–column, and the mechanical resonances obtained by opening or closing tone-holes. The colors of the sounds that will be emitted by the instrumentalists depend on the air attack of the reed involving the lips as well as the pressure and the precision of the airflow. Musicians have also to press their lips on the reed blades to intentionally control the slit opening between the two reeds, in order to tune the color, the pitch, and loudness of the notes that they want to play.

**FIGURE 2:**
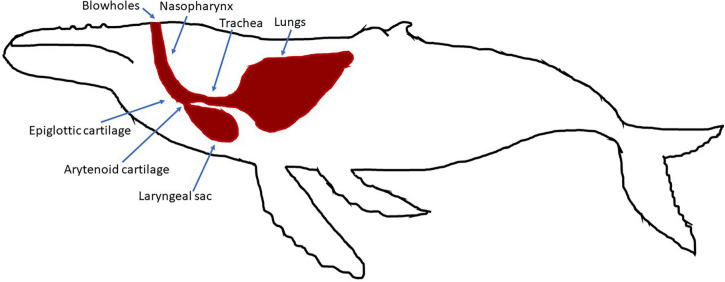
Respiratory system for humpback whales.

The humpback whale vibrator is made by two parallel identical 30-cm-long cartilages, called arytenoid cartilages, covered by a more or less thick membrane over their entire length (Reidenberg and Laitman, 2007; Figure 2). The arytenoids work as a valve. The whale will unseal these cartilages by relaxing the muscles so that air can move inside the respiratory system. The whale can move the cartilages in three directions: (a) to longitudinally open with a constant gap on the whole length, (b) to gradually open them in triangular form larger at the apex, or (3) to place them in a V shape (Damien et al., 2018). By adjusting the slit opening of the cartilages, and also by adjusting the stiffness of the membranes and the pressure from the airflow, the whale can precisely control the acoustic features of the emitted vocalizations that allow a great variety of sounds to be generated (Adam et al., 2013; Damien et al., 2018).

The humpback whale vibrator therefore has no common anatomical characteristic with the vocal folds of terrestrial mammals and monkey lips of the odontoceti species. The arytenoids cannot be stretched more or less to modulate the frequencies as terrestrial mammals do. As well, their vibrator can produce pulsed sounds but no clicks as odontoceti species do. The functional anatomies of their own sound generators explain the different time and frequency acoustic features: the clicks emitted by odontoceti species are transient broadband signals, and the humpback whale vocalizations are tonal signals.

Even if the vibrators of the bassoon and of the humpback whale vocal generator are far from each other in terms of the intrinsic features of the materials, including the size, the stiffness, and the thickness, the way to vibrate follows the same principle of fluid dynamics: the specific volume and speed of the airflow creates pressure and passes through the opening between the two constrained symmetrical rigid parts. These two systems can be both modeled by a harmonic oscillator as the mass (*m*_*s*_)–damper (*r*_*s*_)–spring (stiffness *k*_*s*_) controlled valve:

$$m_{s}\,\frac{\partial^{2}z}{\partial t^{2}}+r_{s}\,\frac{\partial z}{\partial t}+k_{s}\,\left(z-z_{0}\right)=p_{r}-p_{m}$$

where *p*_*r*_ and *p*_*m*_ are the inner and outer air pressures, respectively. For the bassoon, *p*_*m*_ and *p*_*r*_ are the pressures before the reeds from the mouth and after the reeds, respectively. For the whale, these two pressures are, respectively, in the trachea before the arytenoids and right after the arytenoids at the entrance of the laryngeal sac.

In both cases, we considered the flows as low as possible to be laminar, which allows to simplify the equation and obtain preliminary simulated results from physics models (Almeida et al., 2003). Note that non-linearities in vibrator oscillations, especially due to faster variations of the airflows, are usually smoothed by the mass damper in the physics models (Almeida et al., 2003; Guillemain, 2004). However, whether for the current playing of the bassoon or for the loud vocalizations emitted by humpback whales, the airflows have to be mainly considered as turbulent due to the high volumes of air relative to the size of the duct and the resulting pressures on the vibrator walls (Wijnands and Hirschberg, 1995; Adam et al., 2013).

**FIGURE 3:**
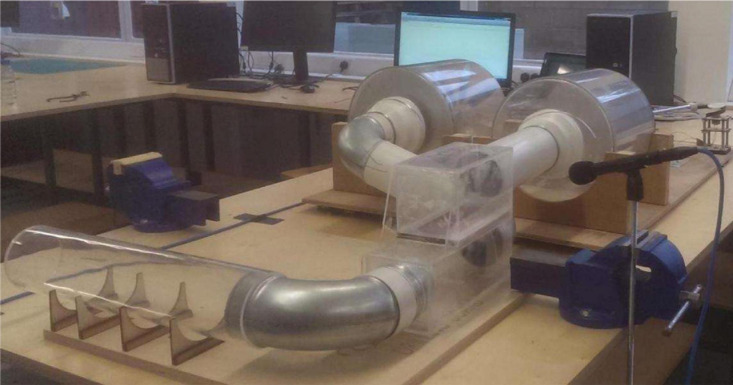
Mechanical model of the humpback whale vocal generator.

Feedback controls (from the instrumentalist and the whale) make it possible to adjust the resonance of the vibrators and to sustain the harmonic oscillations. Bassoon players will more or less force on his/her lips to slightly modify the *p*_*m*_ values. For the whale, a fat membrane called the cushion is localized perfectly above the arytenoids. Its role is to dramatically reduce the space just before the vibrated membranes covering the arytenoid cartilages (Damien et al., 2018). Basically, this cushion brings the airflow to the arytenoids and also presses the membranes around the cartilages, in the same way that the bassoon instrumentalist does with his/her lips on the reeds. An original mechanical model of the humpback whale vocal generator was designed and used to test the different pressures on the vibrator and also to characterize the airflow propagation especially before and after the arytenoids (Lallemand et al., 2014; Cazau et al., 2016; Midforth et al., 2016; Figure 3).

### From Structure and Anatomy

Interestingly, geometrical similarities were also found in the structure and the size of both sound systems.

The bassoon is made with a 2.50-m-long conical drill tube made of precious wood (Figure 1). The total length is 1.30 m when they are folded up. The internal pipe is conical with a diameter that widens regularly from 4 to 40 mm. It is composed of four parts: the wing joint, the butt, the bass joint, and the bell. Depending on the construction, about 30 tone-holes are positioned on the instrument. A curved bocal, a 30-cm metal tube, connects the instrument to the mouthpiece. The original compass was 3 octaves upward from the B*b*0 (58 Hz). However, the modern version now allows to reach treble E*b*5 (622 Hz). The sound timbre also changes by opening and closing the tone-holes.

The humpback whale vocal generator is also made with two pipes linked by the arytenoids: the trachea and the nasal cavities (Figure 2; Reidenberg and Laitman, 2007; Adam et al., 2013; Adam et al., 2018). The trachea is the pipe between the lungs and the arytenoid cartilages. The nasal cavities and the laryngeal sac are the acoustic resonators. The features of these two pipes vary from one individual to another, with lengths from 0.60 to 1.20 m (Reidenberg and Laitman, 2007). This specific anatomy allows them to produce tonal sounds with a fundamental frequency less than 100 Hz. To produce sounds, humpback whales can include or not the nasal cavities by opening or closing the epiglottis. Then, the nasal cavities focus more energy in specific harmonic frequencies, called formants, that can be up to 8 kHz (Adam et al., 2013).

### Temporal Aspects of Sounds

Unit repertoires are not easy to create, especially because of the variabilities of the unit features, even for repetitive units emitted by the same singer. Therefore, manual annotations of acoustic datasets, even by experts trained to detect and classify units, showed inter-annotator variability for different reasons, and in particular due to the subjectivity of the analysis (Leroy et al., 2018). The automatic approaches clearly help to better describe these songs. A very large panel of methods were proposed, for example, based on the information entropy (Suzuki et al., 2005), on a threshold on the Fourier coefficients (Mellinger, 2005), on the extraction of the edge contour (Gillespie, 2004), on the analysis of the mel-frequency cepstral coefficient (MFCC) (Pace et al., 2010) or the wavelet coefficients (Kaplun et al., 2020), or with the use of artificial neural networks (Allen et al., 2017; Mohebbi-Kalkhoran, 2019). To go further with the perception of these vocalizations, new representations were suggested and were very interesting to better extract the similarities of units in these songs, especially based on colored pictograms (Rothenberg and Deal, 2015). These representations, even though with less precise details, encouraged us to think differently about the vocalizations and the whole time structure of the songs. However, automatic classifications are still a current challenge because whale singers are not predictable like machines and constantly add changes and ornaments in their units, altering the duration, the fundamental frequency, some formants, or even the shape of the vocalizations.

The units’ acoustic features are important, but some of them also seem to play a specific role in the syntactic structures (Mercado III, 2016). Durations of silences between units in the successive phrases give specific rhythms (Schneider and Mercado III, 2018). In this study, we also focused on the intonation and the prosody of the themes, including the duration, the melody, and the rhythm of the successive units (Picot et al., 2007).

Musical sounds can be described in three temporal phases, named attack, sustain, release (ASR): the *attack* when the vibration is enough to generate a sound, the *sustain* when the mechanical control is adjusted to maintain the sound as a stationary process, and the *release* when the energy comes lower and lower. Schaeffer (1967) describes more in detail different types of sound morphologies and further characteristics like mass, timbre, pace, dynamics, and profiles. The attack part plays a specific role in music and human perception of sound events. It is one of the (multiple) central questions of electroacoustic music and *musique concrète*. Schaeffer even suggested to cut off these attack parts in order to no longer be able to identify the musical instrument from which the sound was emitted. The objective is clearly to lose any references about the origin of the sounds and then to consider sound objects.

For humpback whale vocalizations, it was also possible to identify these three classical ASR temporal envelope phases. Attacks are directly correlated to the minimum of the sub glottal pressure needed to excite the arytenoid membranes and to set this vibrator into oscillations (Adam et al., 2013). Then, the sustain part appears when the whales control the vibrations propagated to the laryngeal sac, and to the nasal cavities when opened. They adjust the global shape of the vocalization with static or dynamic amplitude and frequency modulations by reducing the glottal flow intensity and/or by modifying the biomechanical properties of the vibrators, like the surface and the stiffness of the arytenoid membranes. Finally, the whales will decrease the outer pressure, by reducing the air flow from the lungs, until reaching the static inner pressure of the fully inflated laryngeal sac. These decay parts can be highly variable, with short or long time durations.

Our method started from careful listening of the emitted whale songs, which is the first step in showing whales that they are being listened to. Then, our approach is to go further than standard playback studies: firstly, our study is not based on simple synthetic sounds or pre-recorded sounds but on sounds created adaptively and suggested from whale vocalizations in real-time. Secondly, the musical interface we used makes it possible to introduce specific features, as modified attacks of few units in the phrase, or playing with the duration of silences to highlight some units like the pulsed sounds, for example. The interface gave us the potentiality of real two-way interactions.

### Airflow Recirculation

For wind musical instruments, the airflow is used to put the vibrator actively into oscillations. The air is coming directly from the lungs, and the durations of the musical notes are directly linked to the specific apnea abilities of the musicians. Thus, aspects of an individual musician’s performance style are partially determined by their breath control. However, in order to obtain a tonal sound held over a longer time period, wind instrumentalists have developed a particular technique: the circular breath. This technique is based on the use of the air stored inside the mouth, especially in the cheek and jowl cavities. This air volume can be used as a second source of air after the lungs in order to maintain the sustain part or to modulate the current notes based on the unidirectional airflow. The contemporary bassoonists with whom Aline Pénitot works have all explored circular breathing.

Even if humpback whales do not take advantage of their mouth to perform the “circular breath” described previously, it was interesting to notice that they also moved the airflow in two directions inside their respiratory system. The natural one is the way from the lungs to the laryngeal sac. The opposite way is back from the laryngeal sac to the lungs, in a circular breathing process (Reidenberg and Laitman, 2007). The main objective of this bidirectional airflow is to make the apnea longer, but whales could also use this technique to increase the duration of their vocalizations or to add new tonal shapes (Adam et al., 2013).

In both cases (bassoon and humpback whale), airflows are crucial to create the initial vibrations of the oscillators and then to maintain and control the sustainable part of the sounds. These flows can be used with air coming from the lungs or coming from a second tank, as the laryngeal sac for the whale and the mouth cavity for the bassoon instrumentalist. These two options of the air sources could increase the acoustic diversity of the expected sounds under the mechanical and anatomical constraints of these generators.

### Pulsed Sounds

Musicians also work the instrument in all of their mechanical and musical capacities, assembled or disassembled. For example, by blowing directly into the bell (the upper part of the bassoon) or the boot (lower part), certain types of bassoon sounds are obtained, or by blowing with a reed pierced in the boot, pulsed sounds can be produced. These musical sounds are patterned in time sequences of successive tonal or transient sounds, spaced enough to be distinguishable by human listeners for which the time separation hearing efficiency is around 50 and max 100 ms (Auriol, 1986).

Humpback whales are also able to generate this type of sounds. Pulsed sounds are emitted when they simultaneously release the arytenoid membrane stress and reduce the airflow through the vibrator but keep it strong enough to maintain the membrane in oscillation. It is interesting that similar pulsed sounds can be found in human speech, as the glottalization, popularly known as vocal fry (Childers and Lee, 1991; Redi and Shattuck-Hufnagelf, 2001).

For bassoons and humpback whales, pulsed sounds can finally be obtained under similar biomechanical conditions, by slowing down the movements of the vibrators. In both cases, these vibrators can produce two different types of sounds: tonal and pulsed sounds. The range of both vibrators extend to the production of chaotic sounds (Cazau and Adam, 2013; Cazau et al., 2016).

## Methods and Results

From our “*trouvaille*,” we were able to design sound objects. This process was motivated by decorrelating the sounds from their origin (in our case, from the bassoon that produced them), in accordance with Schaeffer’s approach. The use of the bassoon was needed to better work on the similarities described previously, but it should not be a limit to the perception of the sounds, as labeled as bassoon sounds. The way to envision *musique concrète* is to go beyond the instruments—or the things that create the original sounds—so as not to be limited by its mechanical constraints and also by the representation listeners can have from it (Schaeffer, 1966).

### Production of Concrete Sound Elements

Considering the acoustic and morphologic similarities between the bassoon sounds and the humpback whale vocalizations, sessions in a professional music recording studio were planned to elaborate and to record a typology of sound morphologies with special attention to the attack, described previously (see section “*Temporal Aspects of Sounds”*), using Schaeffer’s methodology of analytic listening and characterization of sound objects. These recorded sounds are then classified and renamed by their morphological and typological characteristics. This approach is called reduced listening by Schaeffer. “The sound is listened to for itself, disregarding the real or supposed origin, and the meaning that it can convey” (Chion, 1983). We applied this approach to create a library of 47 sounds (available to readers by request to the corresponding author) originally produced with the bassoon and played taking into account the acoustic features of the units emitted by humpback whales. Following Schaeffer’s method, our sounds were classified and named in the rest of the article as *concrete sound elements* (CSEs). To name them, onomatopoeias were used (*Waou*, *Oyé*, and *Ouin*), characteristics of intention (accent and support), or a variation around the notion of pulsed sounds (*Frot*, iteration, and shuffling). The first word used to name the sound is the main characteristic, and then secondary characteristics are added depending on the quality of the sound. The purpose of these CSEs was to broaden the perception of the whale units. This unique sound library was used during human–machine–whale interactions.

### Data Collection

In their study of humpback whale songs, Payne and McVay (1971) conclude that “the function of the songs is unknown.” However, because singers were recorded during breeding seasons, it was firstly assumed that songs could be linked with mating activities, as interactions between females and males, and/or between males. Up to now, the role of these songs is not totally clear and still under investigation. It is the same for the time structure and the meaning of its evolution over the years. Therefore, acoustic playbacks on humpback whales should be done with high caution. In any case, this kind of experiment has to be done by experts, with marine mammal observers (MMOs) on boats and following the rules of protection and respect of the cetacean species.

Therefore, this project done during the humpback whale breeding season in July–August 2018 off La Réunion Island (Indian Ocean) followed strict ethics rules, especially based on the charter of responsible approach of marine mammals (Region Réunion, 2017). During the three field missions, our boat was stopped at 300 m away from the isolated singer, with the engine turned off. No swimmers or divers went into the water. A MMO was on board to observe behaviors of humpback whales and to report any exterior signs from the whales that could be interpreted as a disturbance or a harassment, like stop singing, change in the breathing rate, moving away from the boat, or any exhibition of what can be perceived as agonistic behaviors.

After the experiments, humpback whales were not tracked. The boat went away at very slow speed (3 knots) up to 500 m before adopting a higher speed.

**FIGURE 4:**
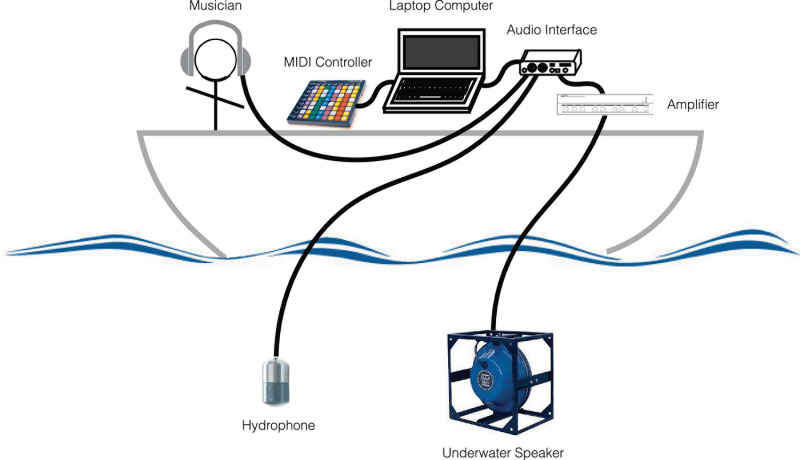
Play and recording setup for the 2018 interactive data collection.

During the experiments, the sounds were played by the musician-experimenter seated on the boat desk, using a laptop and a MIDI controller. The hydrophone Ambient, ASF-1 MKII, and the underwater speaker Lubell LL916 were deployed in the water at 10-m depth right under the boat (Figure 4). The acoustic intensities were lower than 135 dB re 1 μPa at 1 m. The duration of the experiment was limited to 15 min and was done just one time to the same whale.

Before any sounds in the water were played, the humpback whales were visually observed and their songs were carefully listened to. Then, to initiate the whale–human interactions, the concrete sound elements were chosen in the sound library taking into account the vocalizations emitted by the humpback whale singer. Because the CSEs were recorded in a music studio before going on sea, the approach of the musician-experimenter was not to mimic the humpback whale units but more to play CSEs that could match some acoustic features of units, taking into account the musical structures of the phrases. These CSEs were played in real-time, using the sampler connected to the underwater speaker following, anticipating, and synchronizing the successive units, in order to adjust the rhythm and tonation.

For this study, we analyzed the acoustic recording done during the last experimental day because (a) it corresponded to the longest interaction, (b) the recording was done continuously with no interruption, and (c) the weather conditions and the sea state were sunny and calm.

The duration of this acoustic recordings (48 kHz, 24 bits) was 17 min 35 s. During the annotation phase, the bioacoustic expert manually detected all units and concrete sound elements, by carefully listening to the acoustic recordings and by visually examining the spectrograms. The types of the sounds (tonal and pulsed; Adam et al., 2013), the contour of the fundamental frequency (flat, upsweep, downsweep, convex, concave, and continually modulated shape; Hickey et al., 2009), and the presence of harmonic frequencies (Adam et al., 2013) were used to create new classes. When only the unit durations were significantly different but the other acoustic features were still close, sub-classes were created and notated with the letter of the class and a number (for example, C2, C3, and C4 are subclasses of C). In a few cases of overlapping units and CSEs, they did not start at the same time, and it was easy to manually classify them. Finally, for this acoustic recording, 307 acoustic events were detected, including 40 concrete sound elements and 267 units. To name the units, we used letters of the Latin alphabet, assigned in alphabetic order, for each new type of vocalization successively detected in the acoustic recording, as suggested by Payne and McVay (1971). The same letter followed by a number is used when the units are closed enough not to create a new category. Finally, the humpback whale individual emitted 21 different units, and 13 different concrete sound elements were played during this experiment (Table 1). These units and CSEs are available to readers by request to the corresponding author.

**TABLE 1:** Names and occurrences of the humpback whale song units (SUs) and the played concrete sound elements (CSEs).

SU		CSE		
Notation	Occurrence	Name	Notation	Occurrence
A	2	Oye Canard 2	OC2	1
B	6	Oye Canard 3	OC3	1
C	41	Pulsed 1	P1	4
C2	57	Plaint 1 double	P1D	3
C3	3	Plaint 2 double	P2D	1
C4	8	Pulsed 2 frot	P2F	2
D	64	Plaint 4 ouin	P4O	2
E	2	Pulsed 5 appui	P5A	2
F	2	Plaint 5 deux ouins frot	P5DOF	12
H	2	Plaint plus 1	PP1	6
I	1	Plaint plus 3 appui	PP3A	1
J	17	Waou frot 1	WF1	1
K	2	Waou frot 1 double	WF1D	1
M	4	Waou frot grav 1	WFG1	2
O	11	Waou frot grav 2	WFG2	1
P	19			
P2	9			
P3	8			
Q	2			
Q2	3			
R	4			

### Comparison Between Concrete Sound Elements and Humpback Whale Sounds

**FIGURE 5:**
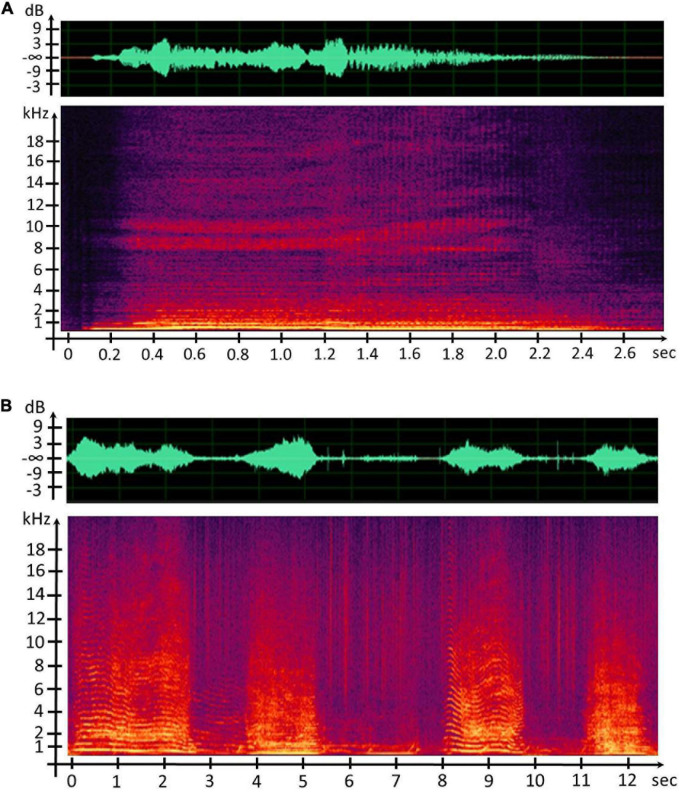
**(A)** Waveform and spectrogram representation of the concrete sound element (CSE) “Waou frot grav 2.” **(B)** Waveform and spectrogram representation of humpback whale song units.

After mechanical and anatomical similarities between the sound generators were noticed, the underlying question was to know if the CSEs and units share common acoustic features or at least were distributed close to each other. We firstly provided the spectrograms using the 512-sample fast Fourier transform and the weighted Blackman–Harris window, with 75% overlap in order to detect all the acoustic events, CSEs, and units (Figure 5).

To compare the CSEs and the units, two different sets of acoustic features were automatically provided with the motivation to investigate two levels of complexity. Firstly, five characteristics were computed: the duration and the spectral properties, including minimum, maximum, peak, and bandwidth. For each feature, the obtained results with the means and the standard deviations are given in Table 2.

**TABLE 2A:** Dispersion of the acoustic features for CSE.

	Frequency (kHz)				Time (s)	Pair-wise Euclidean distance
Notation	Min	Max	Bandwidth	Peak	Duration	
OC2	0.10	5.50	5.40	0.86	0.4	NC
OC3	0.11	4.98	4.87	2.08	0.4	NC
P1	0.17 ± 0.03	2.45 ± 0.48	2.27 ± 0.48	0.80 ± 0.15	2.0 ± 0.1	1.07 ± 0.47
P1D	0.30 ± 0.14	2.34 ± 0.66	2.04 ± 0.71	0.56 ± 0.20	2.8 ± 0.9	1.90 ± 0.38
P2D	0.19	2.82	2.63	0.97	2.8	NC
P2F	0.19 ± 0.05	3.03 ± 0.37	2.84 ± 0.32	0.97 ± 0.02	2.1 ± 0.2	0.79 ± 0.0
P4O	0.09 ± 0.01	3.00 ± 1.23	2.90 ± 1.24	0.77 ± 0.3	1.2 ± 0.0	2.58 ± 0.0
P5A	0.10 ± 0.01	2.10 ± 0.04	2.00 ± 0.05	0.15 ± 0.02	2.1 ± 0.1	0.14 ± 0.0
P5DOF	0.18 ± 0.05	3.88 ± 1.50	3.70 ± 1.54	0.82 ± 0.13	2.8 ± 0.8	2.47 ± 2.17
PP1	0.22 ± 0.06	2.81 ± 0.12	2.59 ± 0.13	0.91 ± 0.48	1.7 ± 0.6	1.09 ± 0.75
PP3A	0.11	2.48	2.37	0.26	0.4	NC
WF1	0.18	2.59	2.40	0.63	3.1	NC
WF1D	0.14	2.78	2.64	0.82	1.6	NC
WFG1	0.17 ± 0.07	2.16 ± 0.15	1.99 ± 0.23	0.45 ± 0.40	2.9 ± 2.6	0.21 ± 0.0
WFG2	0.49	2.46	1.97	0.65	2.6	NC

*Mean ± SD is given for each feature. In the last column, NC (Not Computed) is written when only one sound was in the category. CSE, concrete sound element.*

**TABLE 2B:** Mean and dispersion of the acoustic features for units.

	Frequency (kHz)				Time (s)	Pair-wise Euclidean distance
Notation	Min	Max	Bandwidth	Peak	Duration	
A	0.40 ± 0.07	1.99 ± 0.15	1.59 ± 0.14	0.52 ± 0.02	1.8 ± 0.4	0.34 ± 0.0
B	0.22 ± 0.02	2.03 ± 0.28	1.81 ± 0.27	0.29 ± 0.02	1.3 ± 0.4	0.59 ± 0.25
C	0.12 ± 0.03	1.46 ± 0.70	1.35 ± 0.69	0.18 ± 0.06	0.4 ± 0.2	1.23 ± 0.82
C2	0.10 ± 0.03	2.16 ± 1.12	2.05 ± 1.11	0.18 ± 0.16	0.9 ± 0.4	1.70 ± 1.76
C3	0.35 ± 0.03	2.31 ± 0.05	1.96 ± 0.07	0.41 ± 0.03	0.6 ± 0.2	0.20 ± 0.04
C4	0.27 ± 0.03	2.42 ± 0.25	2.15 ± 0.24	0.35 ± 0.05	2.4 ± 1.0	0.84 ± 0.38
D	0.46 ± 0.06	2.30 ± 0.45	1.84 ± 0.44	0.58 ± 0.04	2.2 ± 0.8	0.99 ± 0.60
E	0.21 ± 0.01	2.79 ± 0.05	2.58 ± 0.05	0.88 ± 0.11	2.2 ± 0.3	0.19 ± 0.0
F	0.08 ± 0.01	2.17 ± 0.18	2.09 ± 0.19	0.15 ± 0.02	1.4 ± 0.2	0.41 ± 0.0
H	0.13 ± 0.04	3.97 ± 1.28	3.84 ± 1.31	0.88 ± 0.40	2.6 ± 0.3	2.70 ± 0.0
I	0.09	2.30	2.21	0.17	1.4	NC
J	0.12 ± 0.03	2.13 ± 0.49	2.01 ± 0.48	0.29 ± 0.14	3.7 ± 1.0	0.92 ± 0.70
K	0.19 ± 0.01	2.88 ± 0.25	2.69 ± 0.24	0.72 ± 0.10	2.7 ± 0.7	0.82 ± 0.0
M	0.22 ± 0.09	2.35 ± 0.40	2.13 ± 0.46	0.32 ± 0.07	2.2 ± 1.8	1.67 ± 0.62
O	0.09 ± 0.01	2.85 ± 0.52	2.76 ± 0.52	0.16 ± 0.01	1.4 ± 0.2	0.83 ± 0.70
P	0.27 ± 0.13	2.75 ± 0.62	2.48 ± 0.68	0.77 ± 0.54	0.6 ± 0.2	1.91 ± 1.07
P2	0.15 ± 0.02	3.24 ± 0.54	3.09 ± 0.55	0.50 ± 0.43	1.6 ± 0.3	1.36 ± 0.86
P3	0.25 ± 0.16	2.80 ± 0.73	2.55 ± 0.83	1.19 ± 0.68	0.8 ± 0.5	2.30 ± 1.11
Q	0.19 ± 0.05	2.72 ± 0.08	2.53 ± 0.13	0.78 ± 0.01	1.4 ± 0.1	0.46 ± 0.0
Q2	0.20 ± 0.03	2.74 ± 0.02	2.54 ± 0.04	0.80 ± 0.03	1.5 ± 0.3	0.33 ± 0.11
R	0.74 ± 0.15	3.08 ± 1.15	2.34 ± 1.20	1.96 ± 0.10	0.3 ± 0.1	2.42 ± 1.15

*Mean ± SD is given for each feature. In the last column, NC (Not Computed) is written when only one sound was in the category.*

The humpback whale units last from less than 1–5 s, as do almost all the CSEs (Table 2B). Only the CSE “WFG1” lasts more than 5 s, and finally, it was the sound with the longest duration (Table 2A). Moreover, regarding spectral properties, these sounds also show similarity, for all CSEs compared with the units. Therefore, the minimal frequencies of the CSEs and the units were between 80 Hz (units “F” and CSEs “P4O”) and 500 Hz (units “D” and “A” and concrete sound element “WFG2”). Only for the unit “R” was this frequency higher than for the other sounds (740 Hz). The peak frequencies were also distributed in the same range, except for the CSE “OC3” (2 kHz) in which we can recognize the resonance property of the bassoon.

**FIGURE 6:**
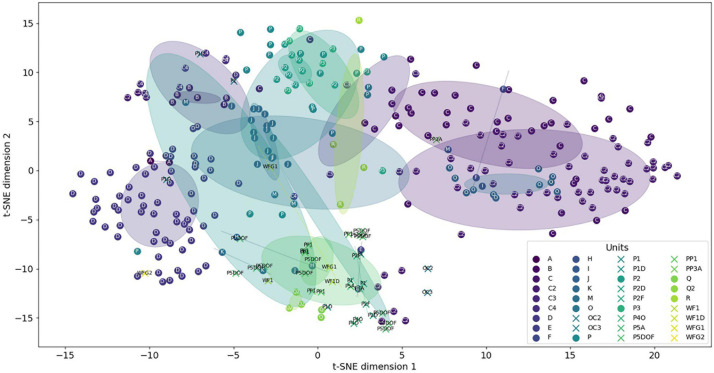
Distribution of the concrete sound elements (stars) and the units (dots). Circles are centered on clusters with a radius corresponding to the standard deviation of 1.

To have a better view, a principal component analysis (PCA) was computed. The two first PCA components provided 46 and 29% of the variance proportion of the data. This proportion will increase to 94% by adding the third component. However, the 3D figure would have been hard to understand, so we chose to keep only the two components. Figure 6 displays, with the same color, all sounds of the same category showing their dispersion inside their own category. We can see that the majority of the CSEs and the units are grouped between -2 and 2 on both axes, showing close values of their acoustic features. The unit dispersions were measured by computing the mean Euclidean distances between these sounds in their own category (Tables 2A,B). As a result, only five sound categories have a mean distance higher than 2. The distances are lower than 1 for 64% of these categories (23 out of 36), showing that the CSEs and units are well clustered. The circles showed the overlapping of the different categories. It means firstly that some units had acoustic similarities. Furthermore, it seemed that the unit reproducibility depends on the type of sounds with lower standard deviations for tonal sounds than for pulsed sounds. Secondly, the units shared acoustic features close to those of CSEs, as shown with Euclidian distances and standard deviations in Figure 6.

However, the diversities were not equal for the different categories (Figure 6). The lower dispersion suggested that the different units of the same category have closer acoustic features than when the dispersion is higher. These diversities can be explained by the different numbers of the units and also because in the same category, the whale can slightly modify one of the parameters. For example, the time duration of units in one category could be longer or shorter, while the types of the sounds (tonal or pulsed) and the spectrogram shapes are still very similar. As a result, in Figure 6, we can see that the two units “A” and “B” are very close to the unit “C.” On the other hand, the C2 category showed a large dispersion, because these units were emitted with lower acoustic intensities and were shorter (meaning that the mean of the acoustic parameters gave less constant values) and also because the occurrence of these units was higher (Table 1).

Secondly, we would like to investigate a higher complexity of these concrete sound elements and units. Therefore, from each detected unit, a total of 74 acoustic features were automatically computed from the time and spectral representations (see the mathematical definition of these features in Malfante et al., 2018) (Table 3). All implementations were taken from the Librosa toolbox (McFee et al., 2020), the audio_analysis software package (Lerch, 2012), and the pyaudio analysis toolbox (Giannakopoulos, 2015). The t-distributed stochastic neighbor embedding (t-SNE) was implemented to enable the representation and visualization of the feature space (van der Maaten and Hinton, 2012) using the Scikit-learn Python package (Pedregosa et al., 2011). The aim of using t-SNE was to explicitly show the similar and dissimilar sounds from the CSEs and the units based on these acoustic features (Table 2). With this approach, dissimilar points should be further from each other (Poupard et al., 2019). On the contrary, if the CSEs and the units share similar acoustic properties, they should be close in the t-SNE map.

**TABLE 3:** Acoustic features used in the t-SNE algorithm.

Name	Number	Delta
ZCR	1	1
Energy	1	1
Energy entropy	1	1
Spectral centroid	1	1
Spectral spread	1	1
Spectral entropy	1	1
Spectral flux	1	1
Spectral roll-off	1	1
Spectral bandwidth	1	NC
Spectral flatness	1	NC
RMS level	1	NC
Renyi entropy	1	NC
Shannon entropy	1	NC
Spectral kurtosis	1	NC
MFCC	13	13
Chroma	13	13

*NC, not computed; t-SNE, t-distributed stochastic neighbor embedding; ZCR, zero-crossing rate; RMS, root mean square; MFCC, mel-frequency cepstral coefficient.*

Note that default parameters were used to compute the features and to compute the t-SNE with the Scikit-learn implementation.

**FIGURE 7:**
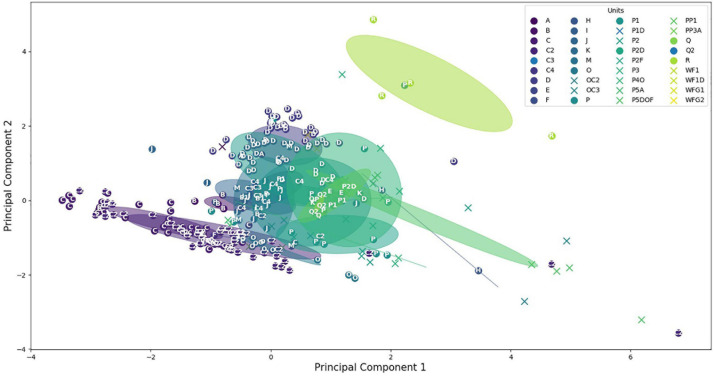
t-Distributed stochastic neighbor embedding (t-SNE) map. Stars represent concrete element sounds, and dots represent humpback whale units. Circles are centered on clusters with a radius corresponding to the standard deviation of 1.5.

From the 74 acoustic features, we also provided the t-SNE map. In Figure 7, we used the same color for all the CSEs and units inside the same category, making it easier to visualize the variability of these sounds. Circles are centered on the cluster of each categories, and the radius was provided for the standard deviation of 1.5.

From this map, we can see that units and CSEs are well clustered showing that the acoustic features enable to discriminate all the different types of sounds. Furthermore, the mixed distribution of the stars, for CSEs, and the dots, for units, shows the close similarity of the different sequences emitted after a specific concrete sound element. For example, the CSEs are found inside the cloud of the units with their t-SNE dimension 1 between −5 and 5 when the units are distributed between −15 and 20. For the t-SNE dimension 2, the CSEs and units share the same range.

We also computed the Euclidean distance between CSEs and units (Table 4). It appears that the mean distance between units is 2.08 and that the Euclidean distances between 2/3 of CSEs and the units are lower than 2.08.

**TABLE 4:** Mean Euclidean distance between the categories of concrete sound elements (columns) and of the units (rows).

	OC2	OC3	P1	P1S	P2S	P2F	P4O	P5A	P5DOF	PP1	PP3A	WF1	WF1D	WFG1	WFG2
A	6.01	5.74	1.46	1.20	1.84	2.17	2.61	1.86	3.33	1.92	2.46	1.39	1.97	**1.03**	1.09
B	5.59	5.72	1.31	1.76	2.07	2.18	1.92	**0.64**	3.39	1.93	1.26	1.42	1.60	0.72	2.29
C	6.35	6.72	2.32	2.84	3.29	3.35	2.90	**1.26**	4.50	3.05	1.59	2.67	2.69	2.01	3.48
C2	5.43	5.83	2.20	2.75	2.89	2.87	2.43	**1.23**	3.92	2.65	1.25	2.38	2.24	1.94	3.43
C3	5.38	5.37	0.92	1.37	1.58	1.76	1.78	0.96	2.98	1.54	1.50	0.94	1.29	**0.19**	1.74
C4	5.17	5.08	0.95	1.33	1.3	1.52	1.79	1.24	2.71	1.34	1.67	0.80	1.18	**0.59**	1.57
D	5.81	5.37	1.65	1.36	1.67	2.03	2.77	2.37	3.13	1.85	2.87	1.45	2.03	1.47	**0.82**
E	4.30	4.07	0.78	1.34	**0.28**	0.53	1.68	1.96	1.75	0.67	2.05	0.41	0.61	1.21	1.61
F	5.26	5.68	1.77	2.33	2.46	2.44	1.94	**0.43**	3.58	2.19	0.47	1.88	1.73	1.37	3.06
H	2.47	2.52	2.50	3.04	1.83	**1.65**	2.30	3.53	2.21	2.11	3.28	2.35	2.09	3.07	3.27
I	5.07	5.47	1.63	2.23	2.28	2.25	1.76	0.41	3.40	2.01	**0.32**	1.72	1.54	1.27	2.96
J	5.30	5.33	1.11	1.57	1.67	1.80	1.80	1.01	3.02	1.62	1.45	1.10	1.32	**0.62**	2.01
K	4.20	4.02	0.87	1.44	**0.50**	0.60	1.60	1.91	1.88	0.82	1.95	0.57	0.57	1.24	1.81
M	5.20	5.26	1.37	1.68	1.81	1.93	1.94	1.16	3.09	1.73	1.45	1.35	1.47	**1.03**	1.96
O	4.29	4.80	1.79	2.45	2.20	2.06	1.62	1.17	3.11	1.95	**0.61**	1.83	1.43	1.70	3.22
P	4.56	4.53	1.48	1.91	1.56	1.58	1.87	1.82	2.70	1.61	1.93	1.35	**1.32**	1.47	2.23
P2	3.68	3.87	1.61	2.24	1.64	1.47	1.47	1.89	2.49	1.61	1.61	1.52	**1.07**	1.81	2.88
P3	4.36	4.16	1.73	2.10	2.03	1.53	2.10	2.46	2.60	1.70	2.54	1.60	1.57	1.91	2.21
Q	4.31	4.29	0.77	1.36	1.19	0.81	1.42	1.52	2.11	0.89	1.54	0.45	**0.26**	0.98	1.95
Q2	4.30	4.24	0.74	1.33	1.24	0.71	1.46	1.6	2.02	0.81	1.63	0.41	**0.28**	1.01	1.87
R	6.16	4.89	4.03	3.72	4.67	3.65	4.78	5.23	4.27	3.73	5.52	3.80	4.18	4.28	**2.80**
Mean	4.91	4.90	1.57	1.97	1.90	1.85	2.09	1.70	2.96	1.80	1.85	1.49	1.54	1.47	2.30

*In bold is the minimum within one row.*

### Pattern Analysis of Stimuli and Humpback Whale Units

Our second objective was to measure the similarities between the time sequences of vocalizations emitted by the whales after the display of a specific concrete sound element. With these comparisons, we would like to show if the whales emit the same sequences or not.

We applied the Levenshtein distance similarity index (LSI) (Levenshtein, 1966), previously used in different studies, especially to assess the changes in humpback whale songs across multiple sites and over years (Eriksen et al., 2005; Rekdahl et al., 2018; Owen et al., 2019). This index allows to evaluate the number of changes needed to transform one song to another. A change could be an insertion, a deletion, or a substitution of one unit in the songs. Furthermore, the representative song (median string) was computed for each stimulus (Owen et al., 2019). In our study, we chose to provide the normalized version of the Levenshtein distance on the unit sequences emitted after each concrete sound element. Thus, we defined each concrete sound element as stimulus (Levenshtein, 1966), and this approach estimated the changes during the sequences of successive vocalizations following the insertion of these stimuli (Table 5). Because we provided distance (distance = 1 – similarity), the 2 compared sequences are identical when the normalized Levenshtein similarity index (NLSI) value is null, and the sequences are totally different when the value is 1. All implementations were performed in Python with the python-string-similarity toolbox (Luo, 2020) with the python-Levenshtein modules (Necas et al., 2019).

**TABLE 5:** Distance index for SU sequences emitted after a CSE.

Stimulus CSE notation	SU sequences emitted after the stimulus	Median Levenshtein index	Normalized Levenshtein similarity index
OC2	“CDDMCCD”		NC
OC3	“C2C2D”		NC
P1	“BCC,” “EBCCD,” “C2C2DC2,” “C2DC2C2D”	“C2BCC”	0.4–1
PP1	“MCCD,” “CCD,” “C2C2C2D,” “C2C2C2D,” “C2DC2C2D,” “C2C2DC2”	“C2C2C2D”	0–1
P1D	“HC4DC4,” “QQ2DRC4C,” “Q2C2C2DROCCDPOC”	HQ2DC2C4C	0.67–0.92
P2D	“CCDMC”		NC
P2F	“BCCDD,” “BCCDFCCDFC2DEC2”	“BCCDD”	0.62
PP3A	“DRC2QQ2DDC2CCCDR C2CCCCDD”		NC
P4O	“IC2D,” “C2C2CDC2C2D”	“C2C2CD”	0.71
P5A	“C2C2DC2,” “C2D”	“C2C2D”	0.5
P5DOF	“C2CCDC2C2CDC2,” “C2CDC2C2CD,” “C2C2DJKJ,” “J,” “JD,” “JDJ,” “JM,” “PDJP,” “PPP2P3P3PP2,” “PPP3P2PP3,” “PP2P3,” “P3P2PC3C3C4P3C3”	“JP”	0.22–1
WF1	“OC2CDOC2CDOC2C2DOC2”		NC
WFG2	“KOC2DOC2DOC2DOC2C2 DOC2C2DJD”		NC
WFG1	“JD,” “DJDJDJDJDJDJDJPPPPP2”	DJD	0.89
WF1D	“P2PPP2HP3PP2PC4 C4DC4DC4”		NC

*NC, not computed; SU, song unit; CSE, concrete sound element.*

The low NLSI values showed that, after the same concrete sound element, the following emitted units have specific acoustic features or the unit sequences are close. After the concrete sound elements PP1, six unit sequences were detected, with the common structure “C2C2C2D” (Table 4). The different NLSI values showed that specific unit sequences can be expected after emitting CSEs.

Two different patterns could be identified for the time unit sequences emitted after the stimulus CSEs “P1” (Table 4). Half of the sequences were similar, including the pattern “BCC,” while the second half always started with the unit “C2.” It included a “C2C2D” pattern and did not include any of the “B” or “C” units. Similar results could be identified in the time sequences emitted after the stimulus CSE “PP1,” which shared close acoustic features with the CSE “P1.” For that stimulus, two unit sequences contained the pattern “CCD,” while the others contained the “C2C2D” pattern instead. This “C2C2D” pattern was also identified in the sequences emitted after the stimuli “OC3,” “P1D,” “P4O,” “P5A,” “P5DOF,” and “WFG2.” The “CCD” pattern was identified after the CSEs “OC2,” “P2D,” “P2F,” “PP3A,” and “P5DOF.”

These results on the diversity of the unit sequences show interesting modifications after the stimuli, which could testify to the singer’s reactions to the CSEs. Nevertheless, this is not sufficient to strictly prove that the stimuli were the only explanation of these changes because sometimes singers repeat phrases quite precisely and other times they do not, even without any playbacks happening. To investigate deeper, further experiments should definitively include acoustic recordings of the whole song before starting playbacks of CSEs, in order to analyze the level of repetitiveness of the unit sequences during the song.

## Discussion

### Acoustic Similarities

It is possible to approach the acoustic features of humpback whale sounds using different types of musical instruments, including singing bowls, bowed, plucked, tapped string instruments, or even human voice. Among all of them, wind instruments are based on vibrators that are close to the vibrator of the humpback whale sound generator, and wind instruments with double reeds are the closest. It takes a high air pressure to obtain a sound with these wind instruments. Therefore, it is very difficult for bassoonists to produce a low-intensity sound. Interestingly, this was also noticed for humpback whale vocalizations (Au et al., 2006). This is explained by the specific anatomy of their vibrator: airflow has to be strong enough to put the membranes that cover the arytenoids in vibration. It depends on the distance between the two arytenoids, their respective positions, and the length and the thickness of the membranes involved in these vibrations.

However, Schaeffer suggested that generated sounds must be distinguished from the musical instrument. Following this approach, the concrete sound elements could be created by using a bassoon or any other instruments or tools. We could assume that acoustic similarities of these CSEs will still be essential to initiate interactions with the humpback whales. The concrete sound elements should not be considered as stimuli, as they were defined for the t-SNE method but they have to be seen as part of the mutual music exchange between the whale and the musician-experimenter. The concrete sound elements have to be played leading or following the humpback whale units taking account of different parameters including the musical timbre, the attacks, the rhythm and the whole structure of the phrases. Using concrete sound elements this way will not be seen as inclusion of new random sounds to disturb the whale during its song, but to suggest sounds that could perceive as musical ornaments inside the original phrase.

Therefore, it will be interesting to renew these experiments and to compare the number of changes in the song phrases during and without the play of the CSEs. This further work could contribute to show whether the whale makes choices in the emission of each unit, of each sequence, or if the humpback singer is primarily following the population-wide song structure.

### Proposal of the Gestural Underwater Interactive Whale–Human Interface

We are not a marine species. Our voice and our musical instruments are not designed to be directly used in the underwater environment. We proposed an interface to tackle this and to allow us to meet the whales in their own local context.

The interface was also the solution to go beyond the human musical instruments that have their own acoustic limits and constraints. The experience gained through our work with playing concrete sound elements with a sampler prompted the design of an original interactive interface. It is motivated by the wish to explore instantaneous interactions between the musicians-experimenters and the humpback whales. The objective is to finely adjust variations of sounds taking into account the variability of the humpback whale vocalizations, with a possibility to create new sounds close to what the musician-experimenter listens to, and also to be at the same level of the whale, in terms of acoustic intensities, frequencies, and variabilities. Thus, this interface is based on simultaneous acoustic recordings and sounds played in the water: firstly, the musician-experimenter are able to listen carefully to the humpback whale vocalizations in order to identify the units, their acoustic features, and also their time structure as sequences, sub-phrases, phrases, and themes. Secondly, the musician-experimenter will choose the adapted CSEs and will play it at the right time inside the humpback whale song, before, after, or simultaneously with the units. With this approach, we expected that the whale would not consider the produced CSEs as echoes of its own units but as sounds that refer to its own vocalizations. Our motivation is not to produce new sounds in the water to humpback whales but to signify to individuals that the musician-experimenter is also here to listen to them and to adjust the choice of the CSEs considering the global time structure of this acoustic exchange. The interface is designed taking into account the four aspects of sound morphology, song structure, intentionality, and gesture. It also allows to signify that silences are listened to.

Up to now, sounds were emitted through a waterproof speaker deployed in the water under the boat, but the musician-experimenter was seated on the boat desk. We thought that the musician-experimenter could also be in the water, for the following reasons: Firstly, with this position outside of the water, the experimenter can feel the border between the marine and air worlds. This could limit the acoustic perception of the produced sounds and also of the vocalizations emitted by the whale. Secondly, the other motivation would be to consider the local soundscape. This is not the same acoustic environment in the water as on the boat, in particular when several people are on the deck, too. Thirdly, to be in the water could be the opportunity to work on the gestures and to link them to how the different sounds are produced. Fourthly, in case of visual contact between the experimenter and the whale, mutual visual observations could be complementary to acoustic perceptions. Even if humpback whale singers are known to be static while singing, the objective is to instantaneously have more information about their potential reactions, postures, and interests: any outside signs could be complementary to their vocal production and could have a specific sense that we need to take into account during this interaction. In the same way, humpback whales could see the experimenter during the performance, could observe gestures, and have information from that. It will be interesting to know how the humpback whales will behave because up to now, studies were done only when humpback whale singers interacted over distances long enough for no visual contacts (Cholewiak et al., 2018). Consequently, a singer that can visualize another agent making song-like sounds and movements at close range is likely to be encountering a radically different scenario from what it would naturally encounter in vocal communication contexts.

**FIGURE 8:**
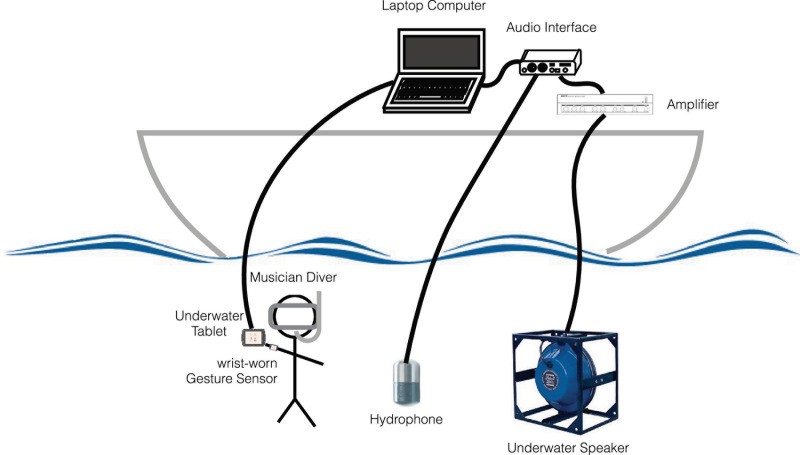
Recording setup with Gestural Underwater interactive Whale–Human interface (GUiWHi).

For all these reasons, we designed our new interface, named Gestural Underwater interactive Whale–Human interface (GUiWHi), to be used also in the water (Figure 8). The components will include the following:

- For underwater audio capture and diffusion, a hydrophone (Ambient, ASF-1 MKII/10 Hz–80 kHz), an underwater speaker (Lubell LL916/200 Hz–23 kHz, 78 W, 180 dB re 1 μPa at 1 m), a diffusion amplifier, an audio interface (RME BabyFace Pro, 24 bits, and 96 kHz with integrated microphone phantom power and pre-amplifier).
- For gesture analysis, sound synthesis and manipulation, and recording of the whole session, a laptop computer (Apple MacBookPro with hard disk, screen, and interactive audio programming environment Cycling’ 74 Max with a bespoke gesture-controlled sound synthesis program).
- For underwater gesture sensing, two wrist-worn inertial measurement units [9-degree-of-freedom (DoF) inertial measurement unit (IMU) Bitalino R-IoT with wired serial connection to the computer, 100 Hz] and one underwater tablet (Valtamer Alltab 4.0 with wired connection to the computer).

The two IMUs allow to finely capture continuous gestures via the built-in accelerometers, gyroscopes, and magnetometers. As such, arm posture (orientation angles), attacks (impulses), and slow arm or forearm movements can be used to initiate and continuously modify sounds, while the tablet with its touch-screen surface allows for preselection of sound classes.

This interface can be played underwater by a diver–musician–experimenter such that a gesture can be associated with a specific type and profile of sound that is produced by the sound synthesis software. This way, a musical development can be elaborated based on careful listening, which is clearly addressed at the whales, going beyond a simple reaction to the type and morphology of their sounds. The musician will sometimes need to take the lead and sometimes let herself/himself be guided. These alternative leaderships between the musician and the whale could potentially provide answers to specific questions about some aspects of these songs, such as why the whale repeats more often specific units than others and what importance should be given to sounds.

## Conclusion

Did we reach our goal to suggest a bidirectional interaction between human musicians and humpback whales? What can we expect from these interactions?

This project is not another attempt to expose one animal species to music or to force whales to listen to human music. The original motivation is totally different, with the idea of starting from their emitted vocal sounds. Our first work was to open our acoustic perception to their songs, based on the large knowledge accumulated from the scientific literature over the last five decades. Then, we would like to better understand the anatomy of their vocal generator, because we thought that coherence and possibilities have to be known before thinking about a complex musical proposal based on multiple unknown acoustic sources. Our goal was to predict if this meeting can work, and what kind of results we can expect in terms of musical exchange. It was very important for this project to stay close to the acoustic features of humpback whales but without imitating them. Finally, we had the opportunity to play our music to one humpback whale individual at the Réunion Island. We observed that the whale stayed around and was not afraid. More of that, it was possible to start musical exchanges taking into account the answers from both sides. On the boat, we were aware about the interaction with this individual whale during this experiment.

This work was to submit a proposal based on *musique concrète* with the objective to create an exchange not based on a competition for vital activities, like protection of their territories, finding mates, defending harems, raising calves, and reactions to predators. The project was initiated to allow opportunities for one-to-one interactions at the same level. It means that our approach was definitely not to lead the music piece and to impose the music orientation. This preliminary study has limitations, especially about how the whale perceived our musical intention. The whale singer may not have been thrilled with this human interjection of concrete sound elements into its performance, even if it tolerated it. This singer was free to change or not the structure of its song and the intonation of its units and, by this way, suggested to the human musician-experimenter to follow these variations or to include new “musical” ornaments.

Perspectives could open inspirations to compose new music pieces in different styles. We also would like to take into account the acoustic characteristics of underwater soundscapes. Indeed, the music perception is influenced by the proportion of biophony, geophony, and anthrophony, and the context in the local geographic sites has to be taken into account during further interactions. It will be very interesting to test a large variety of areas, including shallow waters or underwater canyons. Our idea is also to organize a concert for the public and to observe behaviors in order to describe the perception of what happened and finally their real relationship to nature.

Our work is not a new reason to increase noise in the oceans, although we support the effort of many organizations and institutions that are involved in underwater noise mitigation. Underwater noises are still important, and the effects of these anthropogenic sounds are now well described in the scientific literature. Taking account of this world concern, this project could be seen as an alternative to contribute to cetacean welfare, as music is used as a tool to decrease stress for some animal species (Alworth and Buerkle, 2013; Dhungana et al., 2018), for example, like gorillas (Wells et al., 2006), horses (Eyraud et al., 2019), and domestic species (Hampton et al., 2020; Lindig et al., 2020). Of course, this beneficial effect on stress will have to be proven on humpback whales, for example, by requiring cortisol monitoring (Rolland et al., 2012; Mingramm et al., 2020).

We humans have been able to develop relationships between music from very different cultures and sometimes even with musicians who do not speak the same language and in whose culture music has a very different function. Perhaps, through the process of *musique concrète* and the development of this interface, we will find ways to interact more and more deeply with the whales. This will not happen without further questioning what over the centuries the conventional Western thought has called culture and what we have called nature. Beyond the questions around animal welfare, the acquisition of knowledge and the conservation, the protection, or even the repair of our misdeeds against the environment and animals, it is without doubt that this project must be anchored at the heart of the reflections about nature/culture in order to define peaceful relations between humans and whales.

## Data Availability Statement

The raw data supporting the conclusions of this article will be made available by the authors, without undue reservation.

## Ethics Statement

The animal study was reviewed and approved by Institute Neurosciences Paris-Saclay.

## Author Contributions

AP conceived, managed, and developed this project, composed the music, participated in the field work, and did the field missions at La Réunion Island, Indian Ocean, in 2018. OA did the acoustic recordings of humpback whale songs in Ste Marie Madagascar, Indian Ocean, designed the scientific part, and contributed to the field logistics. About the sounds analyzed in this manuscript, OA did the manual annotation of the different acoustic recordings. DC and PN did the comparison of the acoustic features of the CSEs and the units emitted by the humpback whale. DC and PN developed the source code, which is freely accessible on https://osmose.xyz/. AP designed the GUiWHi device, and OA and DS were involved in the setup of the GUiWHi device. AP and OA wrote the initial manuscript. DS revised, extended, and proofread the manuscript. All the authors gave their final approval for publication.

## Conflict of Interest

AP was employed by the non-profit organization – Compagnie Ondas. The remaining authors declare that the research was conducted in the absence of any commercial or financial relationships that could be construed as a potential conflict of interest.
